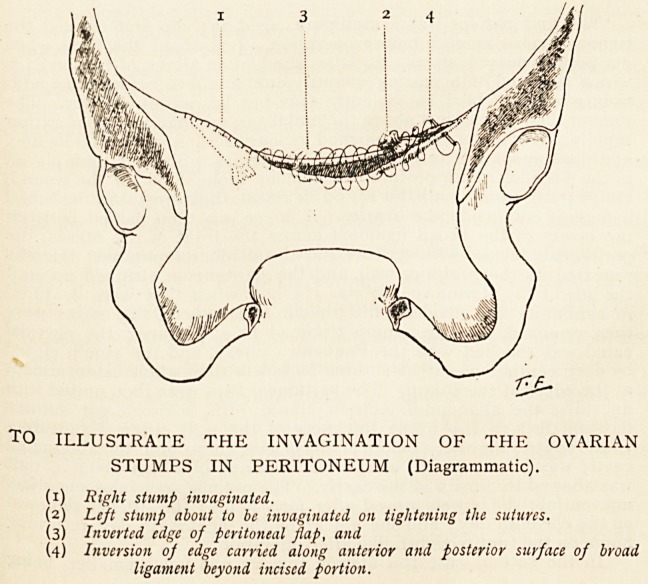# Abdominal Hysterectomy for Fibroids of the Uterus, with Retro-Peritoneal Treatment of the Stump, and Notes of Two Cases
1Read before the Bristol Medico-Chirurgical Society, April 11th, 1900.


**Published:** 1900-06

**Authors:** W. C. Swayne

**Affiliations:** Obstetric Physician to the Bristol Royal Infirmary; Lecturer on Practical Midwifery at University College, Bristol


### ABDOMINAL HYSTERECTOMY FOR FIBROIDS OF THE UTERUS, WITH RETRO-PERITONEAL TREATMENT OF THE STUMP, AND NOTES OF TWO CASES.[1]

BY

W. C. Swayne, M.D. Lond.,

*Obstetric Physician to the Bristol Royal Infirmary;*
*Lecturer on Practical Midwifery at University College, Bristol.*

---

The treatment of uterine fibroids has given rise to considerable divergence of opinion; on the one side we find those who declare that they are innocuous and should be left alone, and on the other those who are of opinion that they should be removed whenever found to be present. Both views are erroneous, but between these two extremes there is a possibility of arriving at a correct conclusion as to the general grounds on which treatment should be based. To take the first contention, that fibroids are innocuous. A short time since a well-known authority asked whether anyone could honestly say that death had ever been caused by a fibroid tumour of the uterus, and added that he could not remember such a case. In a necessarily more limited experience, I am able to state that I know of cases in which death has occurred from conditions brought about by fibroid tumours: in one death occurred from intestinal obstruction; in another from hemorrhage; in another from rupture of the uterus in parturition; and in two cases from malignant disease occurring either as malignant degeneration of a fibroid, or as malignant disease of the uterus already affected with a fibroid tumour. To take the other view. Every obstetrician of any experience has had under his care numerous cases where even large tumours failed to produce any symptoms of importance beyond those due simply to the bulk of the

[1] Read before the Bristol Medico-Chirurgical Society, April 11th, 1900.

tumour; I myself have now under my care several such cases.

We may therefore adopt an opinion midway between the extremes before mentioned, and state that though fibroid tumours of the uterus are often innocuous, conditions may be present which call for operative interference, and these may be briefly stated as follows:—operative interference should be advised (1) if severe hemorrhage is present; (2) if pressure symptoms are present; (3) if in pregnancy the situation of the tumour is such that obstructed labour will result; (4) if severe pain and physical disability arise in working women to such an extent as to incapacitate them; (5) if after the menopause hemorrhage recurs; (6) if the tumour commences to increase with great rapidity, especially after the menopause; (7) if symptoms of twisting of the pedicle, when one is present, should show themselves. The two last are especially unfavourable symptoms.

The operative procedures for the relief of the patient are many; and here again we cannot commit ourselves to any one method without the danger of occasionally omitting to meet the best interests of the patient.

The tumour or tumours may be dealt with by (1) myomectomy, either *per vaginam* or *abdomen;* (2) enucleation through the cervix and vagina; (3) removal of the uterus *per vaginam;* (4) removal of the uterus *per abdomen;* (5) partial removal of the uterus *per abdomen*, with retro-peritoneal treatment of the stump, (6) with extra-peritoneal treatment of the stump; or (7) indirectly by oöphorectomy.

Myomectomy is in a suitable case, *i.e.*, a well-defined pedunculated or sessile tumour, a sound proceeding; it is truly conservative, and, in many cases, has enabled the patient to pass successfully through pregnancy and parturition. It is, however, not devoid of risk, and is quite unsuited for large growths, or those in which uniform enlargement of the corpus uteri is present.

Enucleation after dilating the cervix is most dangerous and should not be attempted in nulliparæ with narrow vagina, pedunculated tumours excepted.

Removal of the uterus *per vaginam* is fairly satisfactory when the tumour is not large and the vagina capacious, it may be performed by morcellation, or in the same way as for malignant disease, but if adhesions be present may be very difficult and risky. Complete removal *per abdomen* (panhysterectomy) is not difficult, and if injury to the ureters can be avoided is fairly safe, especially if the combined method is used.

Partial removal, with retro-peritoneal treatment of the stump, is a satisfactory operation, but has a higher mortality at present than the extra-peritoneal method of treatment of the stump; this, however, we may expect to be corrected as the operation becomes more general. Convalescence in either complete removal, or partial removal with retro-peritoneal treatment of the stump, is rapid, and complications are less likely to occur than when the stump is treated by the extra-peritoneal method.

TO ILLUSTRATE THE INVAGINATION OF THE OVARIAN STUMPS IN PERITONEUM (Diagrammatic).

(1) *Right stump invaginated.*
(2) *Left stump about to be invaginated on tightening the sutures.*
(3) *Inverted edge of peritoneal flap, and*
(4) *Inversion of edge carried along anterior and posterior surface of broad ligament beyond incised portion.*

Oöphorectomy is often unsuccessful, and symptoms due to the sudden induction of the menopause are usually marked.

The two cases reported below were treated by partial hysterectomy, with retro-peritoneal treatment of the stump, and in both the same technique was applied. Special points in the technique followed are: first, the formation of long peritoneal flaps, so as to ensure the covering of all raw surfaces, and the inclusion of the stumps of the ovarian vessels in peritoneum by raising folds from the anterior and posterior surfaces of the broad ligament; secondly, the ligature of the uterine arteries in continuity without involving peritoneum in the ligatures; thirdly, wedge-shaped amputation through the cervix and the closure of the V-shaped flaps by deep continuous catgut sutures; fourthly, the burying of the ovarian stumps within the sutured peritoneal flaps, and the closure of the abdominal wound without drainage, which, if considered necessary, should be made by incising the vaginal vault outside the peritoneum and the use of a gauze drain into the vagina.

The first patient was a multipara, aged 53; she first noticed the tumour twelve months before operation. Just about the time when her periods ceased, six months later, she had an attack of hemorrhage which recurred in increasing quantity until a month before the operation, when she bled so violently that her life was in danger. The tumour then rose to just above the umbilicus, and had increased rather rapidly. Her abdomen was opened by long incision in the middle line, and the tumour felt to be free from adhesions. She was then placed in the Trendelenburg posture, the tumour brought out of the wound, the ovarian vessels and the round ligaments ligatured, and the broad ligaments cut inside the ovaries. A finger was then passed between the layers of the broad ligament across the front of the uterus, the peritoneum incised with scissors and the bladder separated; this was repeated on the posterior wall, and the peritoneum stripped up until the line of the uterine vessels was reached, when they were ligatured in continuity and divided: the tumour and body of the uterus were then removed, and the stump trimmed to a V-shape; the cervical canal was touched with the Paquelin cautery, and the stump closed by deep continuous catgut sutures buried in their whole extent except at the edges of the stump. The peritoneal flaps were then united with fine silk, the abdominal incision closed with silkworm-gut sutures passing through the whole thickness of the wall (after first dealing with a ventral hernia). Before finally closing the wound, the abdominal cavity was irrigated with sterile salt solution, of which about a pint was allowed to remain in the cavity. The patient's convalescence was uneventful; the bowels moved after forty hours; the stitches removed on the eleventh day; and she got up on the eighteenth day, returning home on the twenty-eighth day after operation.

In the second case the uterine tumour was much smaller, being about the size of the fœtal head, with uniform enlargement of the corpus uteri. The same procedure was followed as in the first. It was noticed that every incision was followed by copious hemorrhage, even small vessels in the abdominal wall, hemorrhage from which is usually stopped by pressure, requiring ligature. The patient progressed favourably at first; but at the end of thirty-six hours her pulse began to rise, and she was obviously suffering from hemorrhage. With the assistance of Dr. R. G. P. Lansdown, I re-opened the abdomen and found the pelvis full of blood. The sutures uniting the peritoneal flaps were cut, but no bleeding point could be found, the blood flowing in a general ooze from the uterine surfaces of the peritoneal flaps. Every contact between the stump or flap and a sponge was followed by fresh oozing, and seeing that all the large vessels were secure, the uterine and ovarian stumps being quite dry, I packed the pelvis with iodoform gauze, united the wound after irrigation of the abdomen with sterile saline solution, and put the patient back to bed. The bowels moved freely next day, and flatus was passed naturally, as also at intervals on the following day. When the packing was removed on the third day, the bowels moved again and flatus was passed, but the patient vomited a few times during the day. The next day the patient vomited frequently, but no abdominal distension was present and flatus was passed. The next day sudden collapse and distension occurred, which was relieved by washing out the stomach, but no improvement in the general condition followed. The patient died the following day, the sixth after the operation. On removing the dressings to re-open the wound to check the hemorrhage, I noticed that every suture hole was surrounded by a large ecchymosis, and on cross-questioning the patient found that she was undoubtedly hæmophilic, hence the bleeding. *Post-mortem*, it was found that two coils of small intestine had become interlocked and adherent close to the gauze packing; there was no peritonitis, and but little plastic exudation, except just about the packing and where the two coils had become adherent. The symptoms of obstruction were not definite until the patient's condition was too grave for operative interference.

In the second case, no doubt extra-peritoneal treatment of the stump would have been preferable had the patient's tendency to hemorrhage been known beforehand; as it was, there was no indication that retro-peritoneal treatment was contra-indicated. Panhysterectomy, as an alternative, would probably have been safer, as all the bleeding points could have been ligatured at the time of operation and bleeding from the collateral vaginal circulation thus avoided.

In both cases the indications for operation were clear, the hemorrhage in the first case being severe, and the pain and malaise in the second making the patient a burden to her friends and her life a misery to herself.

## Figures and Tables

**Figure f1:**